# Different aspects of pharmacological heart failure treatment

**DOI:** 10.1093/ehjcvp/pvaf073

**Published:** 2025-11-04

**Authors:** Stefan Agewall

**Affiliations:** Institute of Clinical Sciences, Karolinska Institute of Danderyd, Stockholm, Sweden

**Figure:**
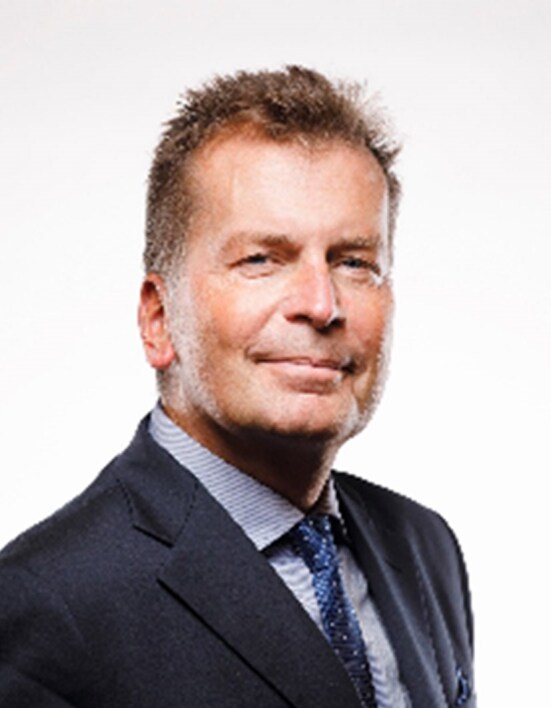


The effect of initiating sacubitril/valsartan (sac/val) therapy during hospitalization for acute heart failure (AHF) on left ventricular (LV) remodelling remains unclear. Dr Tanaka and co-workers from Japan, aimed to assess the impact of Sac/Val on LV remodelling in patients in whom sacubitril/valsartan (Sac/Val) was initiated during AHF hospitalization. The authors used data from Program of Angiotensin–Neprilysin Inhibition in Admitted Patients with Worsening Heart Failure (PREMIER) study, which investigated the impact of initiating Sac/Val during hospitalization for AHF on echocardiographic parameters over an 8-week period, in comparison with the standard renin–angiotensin system inhibitor therapy.^1^

The study group concluded that in patients stabilized after hospitalization for AHF, Sac/Val may significantly improve some functional and structural parameters of the left ventricle, particularly in those with an LVEF < 40%, supporting its role as an effective therapy for reverse remodelling.

Quality of life is an important issue in patients with HF.^2^ Dr McMurray *et al.*, present data on Sacubitril/valsartan and Quality of Life Assessed Using the EuroQol 5-Dimension 3-Level Questionnaire Level Sum Score in patients with HFrEF and HFmrEF/HFpEF. Changes in LSS severity at 8 months were analysed using ordinal logistic regression models to estimate the relative effect of sacubitril/valsartan vs. enalapril or valsartan. The authors concluded that sacubitril/valsartan significantly reduced the risk of heart failure (HF) events and improved health status across the LSS spectrum in HFrEF and HFpEF patients treated with enalapril or valsartan.

While the beneficial effect of beta-blocker (BB) therapy for acute coronary syndrome (ACS) patients with left ventricular ejection fraction (LVEF) < 40% is established, its role in those with LVEF >40% is controversial.^3,4^ Dr Maeder and colleagues from Switzerland, assessed the relationship between BB therapy at discharge and 1-year mortality according to LVEF in a large contemporary ACS cohort (*n* = 7820). The authors found that among patients with LVEF ≤40%, mortality was lower in patients with BB compared to those without (5.9% vs. 14%; *P* < 0.001). In contrast, in patients with LVEF >40%, mortality did not differ between patients with and without BB (2.1% vs. 2.6%; *P* = 0.3). A statistically significant interaction between BB therapy and LVEF stratum was identified.

Angiotensin converting enzyme inhibitors (ACEi) and angiotensin receptor blockers (ARB) are effective and recommended therapies following myocardial infarction with reduced left ventricular ejection fraction.^5^ There is, however, less knowledge on the effectiveness of ACEi/ARBs in those with myocardial infarction with preserved left ventricular ejection fraction (≥50%). Dr Humphreys and co-workers from Sweden used Swedish healthcare registries in more than 16 000 individuals, to emulate a target trial of ACEis/ARBs vs. no ACEis/ARBs for the prevention of a composite outcome among individuals under 75 years with myocardial infarction and LVEF ≥ 50%. The authors used estimated observational analogues of the intention-to-treat effect and the per-protocol effect with confounding adjustment via inverse probability weighting. The estimated risk of a composite of death, myocardial infarction, or HF was similar in recipients and non-recipients of ACEi/ARB. Thus, their estimates suggest ACEi/ARB treatment in myocardial infarction with preserved LVEF does not confer a benefit.

A mineralocorticoid receptor antagonist (MRA) is also recommended in patients with heart failure with reduced ejection fraction (HFrEF) chronic systolic HF.^5^ In a previous nationwide cohort study of more than 14 000 patients with new-onset HFrEF who initiated MRA, no differences in clinical outcomes associated with initiation of eplerenone vs. spironolactone were found. However, side effects were more common among spironolactone users and therefore treatment with spironolactone was more frequently withdrawn.^6^ Finerenone, a newer nonsteroidal MRA, is more selective and has stronger anti-inflammatory and anti-fibrotic effects than eplerenone^7^ and finerenone significantly reduced the risk of all-cause and cardiovascular mortality vs. placebo in patients with type 2 diabetes mellitus (T2DM) across a broad spectrum of chronic kidney disease (CKD) stages.^8^ Dr Tsai and co-workers from Taiwan aimed to evaluate the cardiovascular outcomes in T2DM patients treated with finerenone vs. spironolactone or eplerenone using retrospective cohort analysis in more than 4000 patients. After propensity score matching Dr Tsai reports superior cardiovascular outcomes in patients using finerenone compared with spironolactone and eplerenone in patients with T2DM with significant reductions in major adverse cardiovascular events, mortality, and HF events.

Type 2 diabetes (T2D) is a significant risk factor for the development of HF^5^ and altered lipid metabolism plays a significant role in the development of cardiovascular diseases including HF.^9-11^ Fenofibrate exhibits potent lipid-modifying effects and has demonstrated possible beneficial effects on HF-related outcomes. In the ACCORD trial, fenofibrate, compared to placebo, reduced non-significantly the composite outcome of hospitalization for heart failure (HHF) or cardiovascular death by 18% in people with T2D receiving simvastatin.^12^ In a nationwide cohort study, more than 23 000 patients with T2D (≥30 years), receiving statin therapy were 1:1 matched by propensity score into a statin plus fenofibrate group and statin only group. Dr Kim and co-workers from Korea, investigated the association between fenofibrate use and outcomes of HF in patients with T2D. They found that the addition of fenofibrate to statins was associated with significantly lower risks of HHF and the composite outcome of HHF or cardiovascular death in patients with T2D, suggesting a novel cardiovascular benefit of fenofibrate.

Hyperkalaemia (HK) may be seen in patients with CKD and in HF patients treated with the recommended drugs.^5,13^ The condition might be life threatening, so the problem is highly relevant in the clinic. In this issue of the journal, we are happy to publish a paper entitled: Interdisciplinary recommendations for recurrent hyperkalaemia: Insights from the GUARDIAN-HK European Steering Committee. A panel of nine European experts in the management of HK (four nephrologists, four cardiologists, one internist) reviewed existing guidance and evidence on the diagnosis and management of HK. The panel developed 10 consensus recommendations and a management algorithm across three domains: duty of care, identifying patients at risk of HK recurrence, and managing the risk of HK recurrence.

Men and women are different. The prevalence of a certain disease may differ between the genders, but also the progress of a disease may differ^14^ as well as the response to a certain drug or vaccine.^15,16^ There are sex-related differences (SRD) in body composition and physiology, and in the pharmacokinetics, efficacy, safety and dosing of some cardiovascular drugs. Despite its clinical relevance, the information from randomized controlled trials on SRD on the efficacy, safety, and dosing of cardiovascular drugs is rather limited, sometimes controversial, and generally considered of little clinical relevance. In a review paper, Dr Tamargo and Dr Delpón aim to analyse the reasons and consequences of the limited information on SRD in efficacy, safety, and dosing of clinical practice guidelines recommended drugs (CPGRD), whether the recommended doses are appropriate for women, the differences in the use of CPGRD, and finally, formulate recommendations to close our gaps in knowledge about SRD and reverse the current situation to improve cardiovascular disease prevention and treatment from a sex-specific perspective.
